# Obstetrical Forceps

**Published:** 1849-05

**Authors:** 


*Obstetrical Forceps.*—The reader will observe in this number an account of a newly devised Obstetrical Forceps by Dr. White, Professor of Obstetrics in the Buffalo Medical College, accompanied by a plate. We would commend this proposed instrument to the examination of Obstetricians. It has been submitted to the instrument makers of the city of New York, who unite in pronouncing it, in their view, superior to any other in use. It will be kept by Tiemann, the celebrated instrument maker in that city, and may be ordered there, or at A. I. Mathews, Druggist, of this place.

